# Who’s Who in Zoology: transversal application of gamification and new technologies in university teaching

**DOI:** 10.3389/fvets.2025.1596906

**Published:** 2025-05-09

**Authors:** Jaime Galan-Elvira, Pablo Palau-Irisarri

**Affiliations:** Facultad de Veterinaria, Universidad Alfonso X el Sabio, Madrid, Spain

**Keywords:** gamification, active learning, veterinary education, student engagement, education innovation

## Abstract

**Introduction:**

In today’s hyperstimulating digital environment, shaped by social media and artificial intelligence, education struggles to retain students’ attention. Traditional teaching models risk obsolescence if they fail to adapt to rapidly evolving societal dynamics. However, this context offers an opportunity to innovate while maintaining academic rigor.

**Methods:**

To enhance student engagement and modernize the learning experience, we implemented various gamification tools in the Biology course of the Veterinary Medicine degree. Strategies included a zoology-themed version of the classic game “Who’s Who?”, the use of the Kahoot! platform to review practical content, and interactive sessions designed to promote debate and teamwork in solving complex problems. The impact of these methods was assessed through academic performance data and student surveys focusing on engagement, knowledge acquisition, subject interest, and perceptions of the teaching team.

**Results:**

The introduction of gamified teaching strategies yielded highly positive results. Students reported increased motivation, deeper interest in the subject matter, and a more favorable perception of the teaching team. Moreover, a clear improvement in academic performance was observed, with higher average grades compared to previous years.

**Discussion:**

Our findings support gamification as a valuable tool in higher education, particularly in science-based courses. By aligning teaching methods with students’ expectations and cognitive habits, educators can improve both learning outcomes and classroom dynamics. Gamification offers a means to deliver rigorous content in an engaging format, helping bridge the gap between traditional education and today’s fast-paced, digital society.

## 1 Introduction

Gamification, defined as the use of game elements in non-game contexts (1), is an increasingly valuable tool in higher education. This innovative teaching strategy has been implemented across all educational levels (2) and has proven effective in fostering technical, social, and emotional competencies (3). However, university education continues to face challenges in adapting to a rapidly evolving society while sustaining student interest and engagement (4). Moreover, digital overstimulation among young people has been shown to negatively impact concentration and motivation in educational settings (5).

Gamification has emerged as a strategy not only to enhance motivation but also to improve engagement and facilitate the knowledge acquisition process (6, 7). Notably, it provides accessibility to diverse social groups and promotes inclusivity by fostering engagement and personalizing learning experiences (8). This combination of inclusivity and ease of implementation highlights its potential as a transformative educational approach (2). Despite these benefits, further research is required to assess the long-term impacts of gamification in higher education (9).

Gamification in higher education can be implemented through various strategies to enhance student engagement and learning outcomes. General gamification involves incorporating game-like elements, such as points, badges, and leaderboards, into the course structure to foster motivation and participation by leveraging the intrinsic appeal of games (10). Alternatively, targeted gamification activities, such as interactive storytelling, challenges, or problem-solving tasks, can be used to reinforce specific topics or address learning objectives (10). Tools like Kahoot! exemplify gamification platforms that create interactive and dynamic learning environments, promoting active student participation and engagement through quizzes and discussions (11).

It has been shown to improve comprehension, retention, and motivation in both flipped classroom and traditional teaching models (12). Additionally, Kahoot! supports the reinforcement of key concepts and promotes peer interaction, contributing to a collaborative and enjoyable learning experience (13). These features collectively enhance educational outcomes through gamified strategies.

Generative Artificial Intelligence (GAI) is revolutionizing university teaching by introducing advanced tools that enhance learning environments through personalization, efficiency, and innovation. These systems adapt educational content to individual student needs, fostering engagement and improving performance (14). Real-time feedback and tailored support enable students to learn at their own pace, which is especially valuable in complex subjects (15).

For educators, GAI optimizes teaching efficiency by automating repetitive tasks, allowing them to focus on more complex instructional strategies (16). Additionally, various GAI tools can create interactive and immersive experiences, enriching the quality of instruction and promoting dynamic classroom interactions (15). However, implementing GAI in higher education presents challenges, including ethical concerns about data privacy, biases, and equitable access to technology. Addressing these issues through clear institutional policies is essential to harness the benefits of GAI while minimizing risks (16, 17).

## 2 Materials and methods

The aim of this study was to implement and evaluate various innovative strategies to enhance student motivation and engagement while achieving the educational objectives of the first-year Biology course in the Veterinary degree. To accomplish this, three distinct approaches were employed:

1. The classic game “Who’s Who?” was adapted to the educational content of the Zoology module, using gamified methodologies to teach evolutionary and taxonomic concepts in a more interactive and engaging manner.
2. The widely recognized gamification tool Kahoot! was used as a resource for reviewing practical sessions, offering students a dynamic way to consolidate their learning.
3. Based on student feedback, a dynamic, activity-focused class format was developed and implemented, aiming to further align teaching methodologies with students’ learning preferences and foster an engaging learning environment.

These strategies were designed not only to enhance motivation but also to improve the overall learning experience, providing insights into the effectiveness of gamification and innovative teaching methods in higher education.

GAI tools have been utilized in various aspects of this work. First, relevant bibliographic references were gathered using the online tool *SCISPACE*. *ChatGPT* was employed as an assistant for writing and translation tasks.

### 2.1 Who’s Who? Zoology

The classic game Who’s Who? involves guessing the opponent’s secret character by asking yes-or-no questions, progressively eliminating characters until the correct one is identified. In this adaptation for the Zoology module, 16 evolutionary traits were selected to classify 24 animals into 19 taxonomic groups, following the criteria outlined by Hickman et al. (18). Structuring the inquiry process around these evolutionary traits and the taxonomic hierarchy provides players with a strategic advantage, which can be crucial for winning the game.

**Figure 1:**
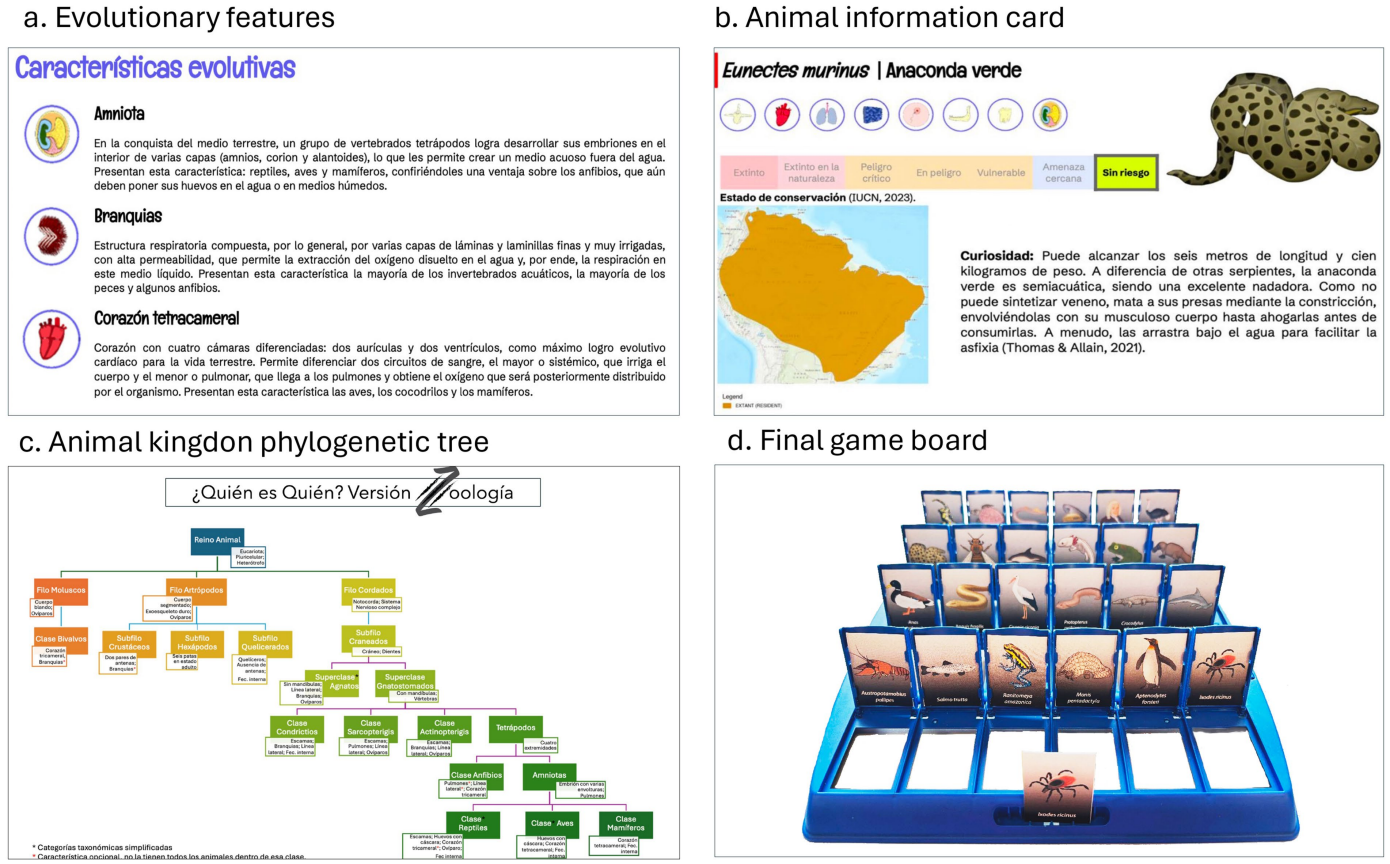
Materials included in the Who’s Who? Zoology! game. **(a)** Evolutionary features. **(b)** Animal information card. **(c)** Animal kingdom phylogenetic tree. **(d)** Final game board.

After selecting appropriate species, a comprehensive script was developed, including concise game instructions and explanations of the evolutionary features present in the game (Figure 1a). This was accompanied by individual cards for each species, which included illustrations, scientific and common names, conservation status, distribution maps, taxonomic characteristics, and scientific fun facts (Figure 1b). Additionally, a simplified phylogenetic tree of the animal kingdom was created for students to use as a “map” of the taxa featured in the game, highlighting the evolutionary traits that position them within the animal kingdom (Figure 1c).

All the representative illustrations of each species and the evolutionary features were created by the authors using Wacom Intuos Comfort S® tablets and software such as Sketchbook Pro® and Adobe Photoshop 2020®.

To measure the impact of the game, online surveys were distributed to students before and after the activity using Google Forms. The surveys included various questions with five response options based on the Likert scale, ranging from 1 (nothing/disagree) to 5 (a lot/strongly agree). A QR code linked to the surveys was provided to facilitate access via mobile devices.

All materials were printed on 300-gram paper, cut to fit the game boards, and inserted into generic Who’s Who? boards (Figure 1d). These customized boards, along with the game manuals and the phylogenetic scheme, were distributed among the students for gameplay.

To explore the potential of GAI, PromeAI, an online tool based on GAI, was utilized. By uploading the original drawings created by the authors, the tool generated new illustrations inspired by the originals. Using Adobe Photoshop 2020®, these AI-generated images were further refined and blended with the original illustrations to produce a hybrid image that was superior to the initial version.

### 2.2 Kahoot as a tool for reviewing content in practical classes

The online platform Kahoot! was used to review key concepts in biology practical sessions. Over the course of three laboratory days, six practical sessions were conducted, with a Kahoot created for each session consisting of five questions, each offering four possible answers.

To assess the effectiveness of this activity, the practical grades of two different cohorts were compared. In the first cohort, Kahoot was not used in any of the practical sessions, while in the second cohort, it was incorporated into all sessions. As a control, the total course grade was used. For data analysis and graphical representation, GraphPad Prism 9 v.9.4.0 software was used, and Student’s t-test was applied. Results were considered significant at *p* < 0.05, *p* < 0.01, and ***p < 0.001.

### 2.3 Dynamic class session

**Figure 2:**
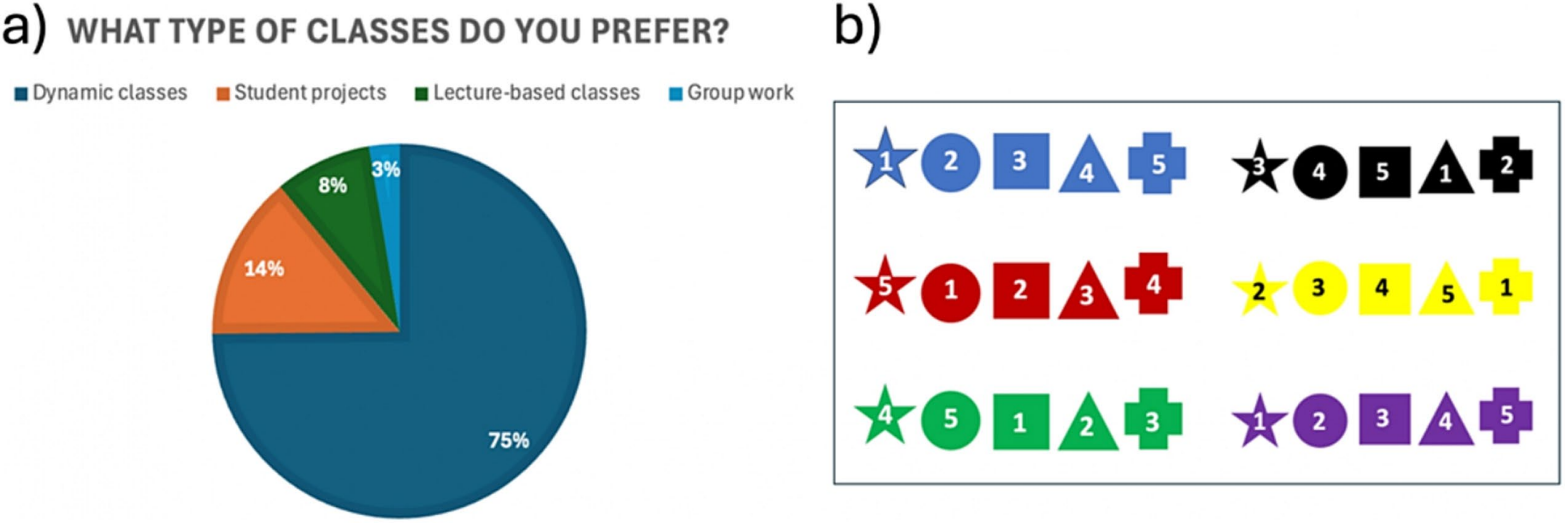
**(a)** Survey results on students’ preferred class types (*n* = 155). **(b)** Tokens designed to create three rounds of randomly assigned student groups based on color, number, or shape.

Based on data collected from surveys conducted with students from different courses regarding their preferred types of sessions (Figure 2a), a dynamic class session was designed. This session aimed to combine theoretical content with soft skills such as teamwork, problem-solving, and public communication in an engaging way.

The class was structured around three problems related to the topics within the biology curriculum: Ethology, Zoology, and Molecular Biology. To solve each problem, students were required to form groups, work collaboratively to develop solutions, and then share their conclusions with the rest of the class through a spokesperson. At the end of each discussion, the teacher analyzed the strengths and weaknesses of each proposal and provided a theoretical and scientific potential solution to each problem.

To encourage participation, students were not allowed to act as a spokesperson more than once. To foster inclusivity and prevent students from staying within their existing friend groups, a token system was implemented to ensure random group assignments (Figure 2b). At the beginning of the session, each student received a token, which determined their group for each problem: colors for the first problem, symbols for the second, and numbers for the third and final problem.

### 2.4 Students’ general perception of the subject after gamification

At the end of the academic year, a survey was conducted to assess students’ perceptions of the biology signature, following the implementation of *Kahoot* in practical sessions, the gamification activity “*Who’s Who? Zoology!*,” and the dynamic classes.

To minimize subjectivity related to affinity toward instructors or gamification activities, students were asked whether the course’s teaching methods aligned with their learning styles. As a control, the same question was posed regarding all university courses, not just Biology.

## 3 Results

### 3.1 Who’s Who? Zoology

**Figure 3:**
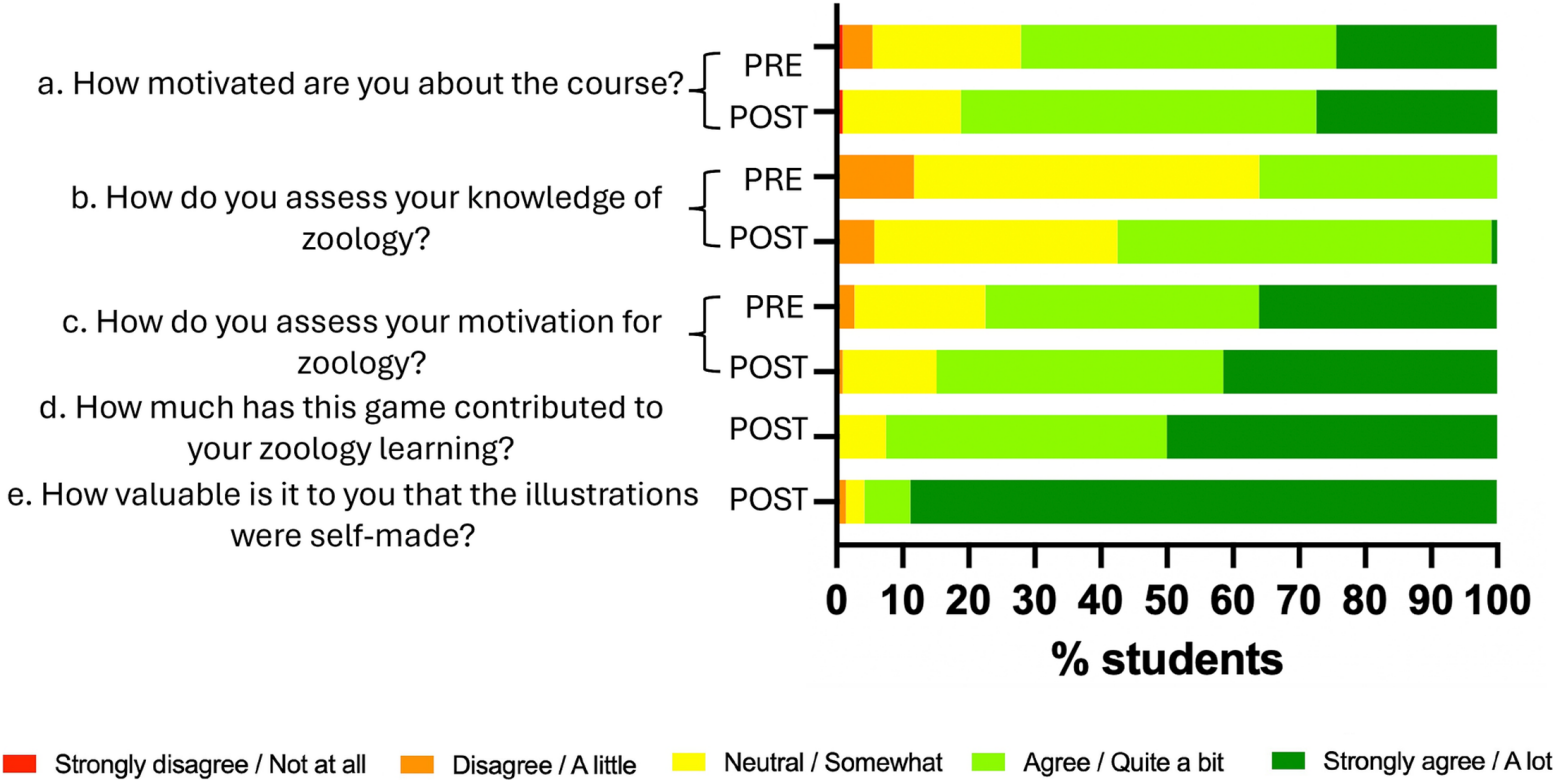
Results of the “Who is Who? Zoology!” surveys. PRE, survey pre-game (*n* = 112). POST, survey post-game (*n* = 109).

The results of the surveys conducted to assess the impact of the activity are presented in Figure 3. Regarding motivation for the academic course, students showed an almost 10% increase in the responses “Quite a bit” and “A lot” (Figure 3b). The most notable increase was observed in the perceived knowledge of zoology, with a 20% rise in positive responses (Figure 3b). Motivation toward zoology also increased after the gamification activity, with a 5% rise in the “A lot” response (Figure 3c). Additionally, 92% of students reported a positive impact on their zoology knowledge due to the game (Figure 3d). Finally, 95% responded positively to the fact that the game’s illustrations were created by the instructors, with 89% selecting the highest rating (Figure 3e).

To assess whether these improvements were statistically significant, a Fisher’s Exact Test was performed. The results indicated a statistically significant association between participation in the game and the improvement in students’ perceptions and motivation, with a *p*-value of 0.006 (*p* < 0.05). This suggests that the gamification activity had a measurable impact on students’ engagement and knowledge in the subject.

**Figure 4:**
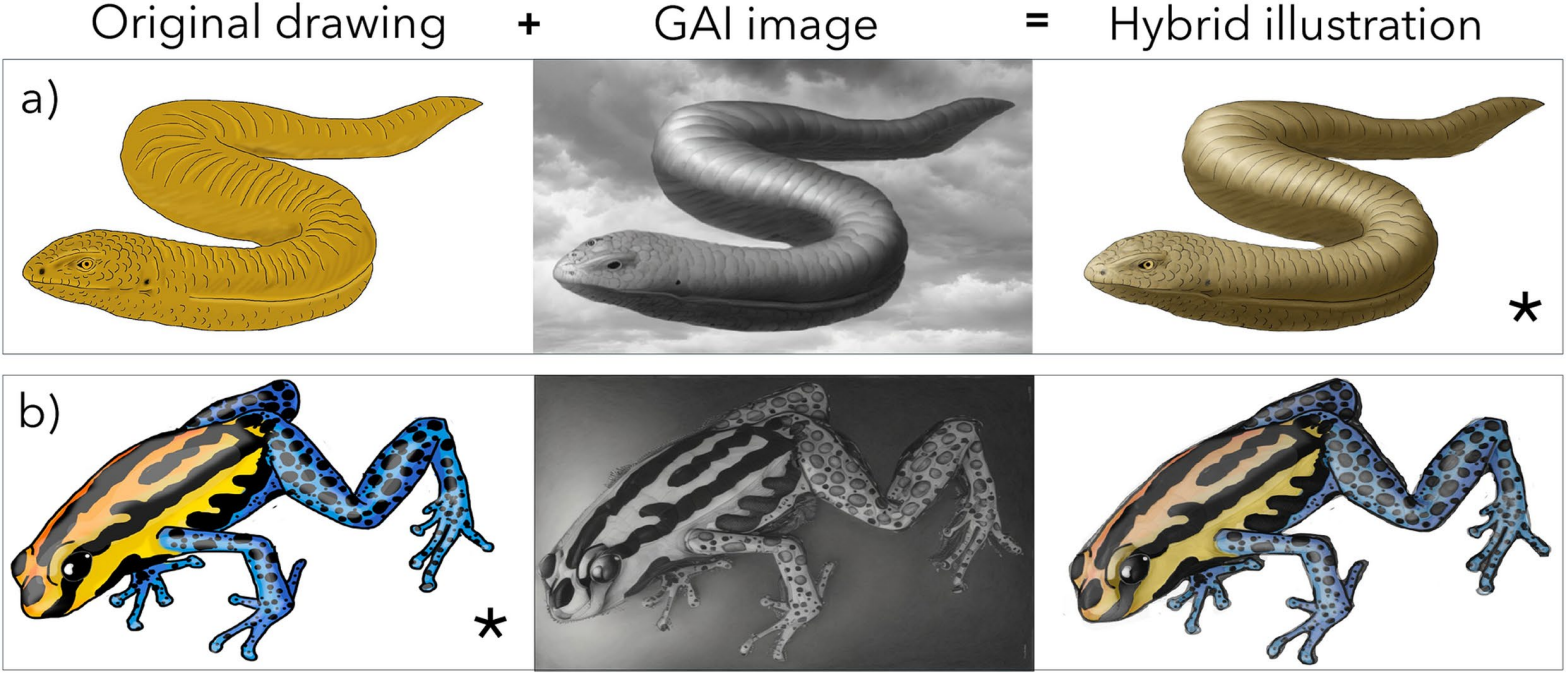
Methodology for using the PromeAI tool to modify the original drawings. **(a)** Original illustration with less time investment and worse results (the asterisk marks the illustrations used for the game). **(b)** Original illustration with greater time investment and better results (the asterisk marks the illustrations used for the game).

Regarding the use of GAI, interestingly, the tool proved effective only in enhancing illustrations that were initially less satisfactory (Figure 4a). In contrast, when applied to higher-quality original illustrations, the AI-generated results led to a decrease in overall quality (Figure 4b).

### 3.2 Kahoot as a tool for reviewing content in practical classes

**Figure 5:**
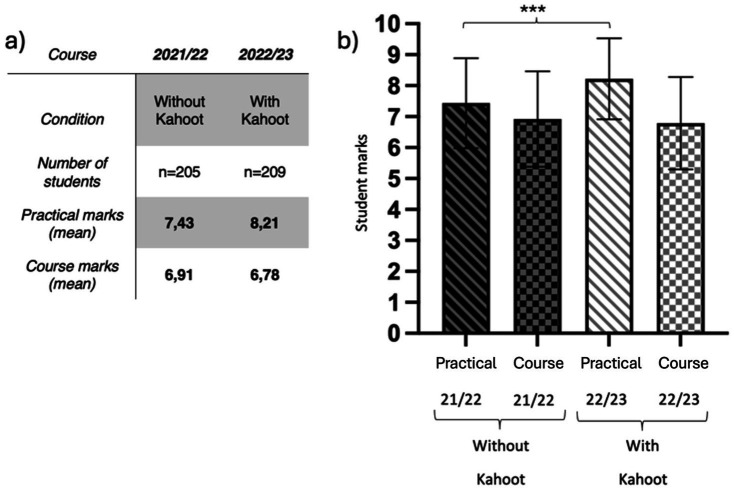
Effectiveness of the Kahoot gamification activity in Biology practical sessions. **(a)** Comparison of practical scores between cohorts with and without Kahoot; **(b)** Graphical representation of the data as mean ± standard deviation. Statistical significance was assessed using Student’s t-test (*p* < 0.001).

The results of comparing practical exam scores between a cohort that used Kahoot and one that did not, with overall course grade as a control variable, indicate a significant increase when Kahoot was implemented, rising from 7.43/10 to 8.21/10 (Figure 5). The control grade for the cohort that used Kahoot was 6.78/10, slightly lower than that of the cohort without Kahoot (6.91). This suggests that the improvement in practical exam performance was not merely due to an overall increase in course grades but was likely enhanced by the specific impact of Kahoot as a learning tool.

### 3.3 Dynamic class session

The dynamic class sessions structured around three core biology topics—Ethology, Zoology, and Molecular Biology—proved to be highly engaging for students. During these sessions, students collaborated in randomly assigned groups to address real-world problems, encouraging active participation. The token system used to assign groups ensured that students interacted with a diverse set of peers.

Each problem-solving session culminated with students presenting their solutions to the class. The instructor provided feedback after each presentation, offering both a theoretical perspective and potential scientific solutions to the problems.

Students reported a greater sense of involvement and engagement, particularly those who typically struggle with socialization, as they were paired with classmates outside of their usual social circles.

### 3.4 Students’ general perception of the subject after gamification

**Figure 6:**
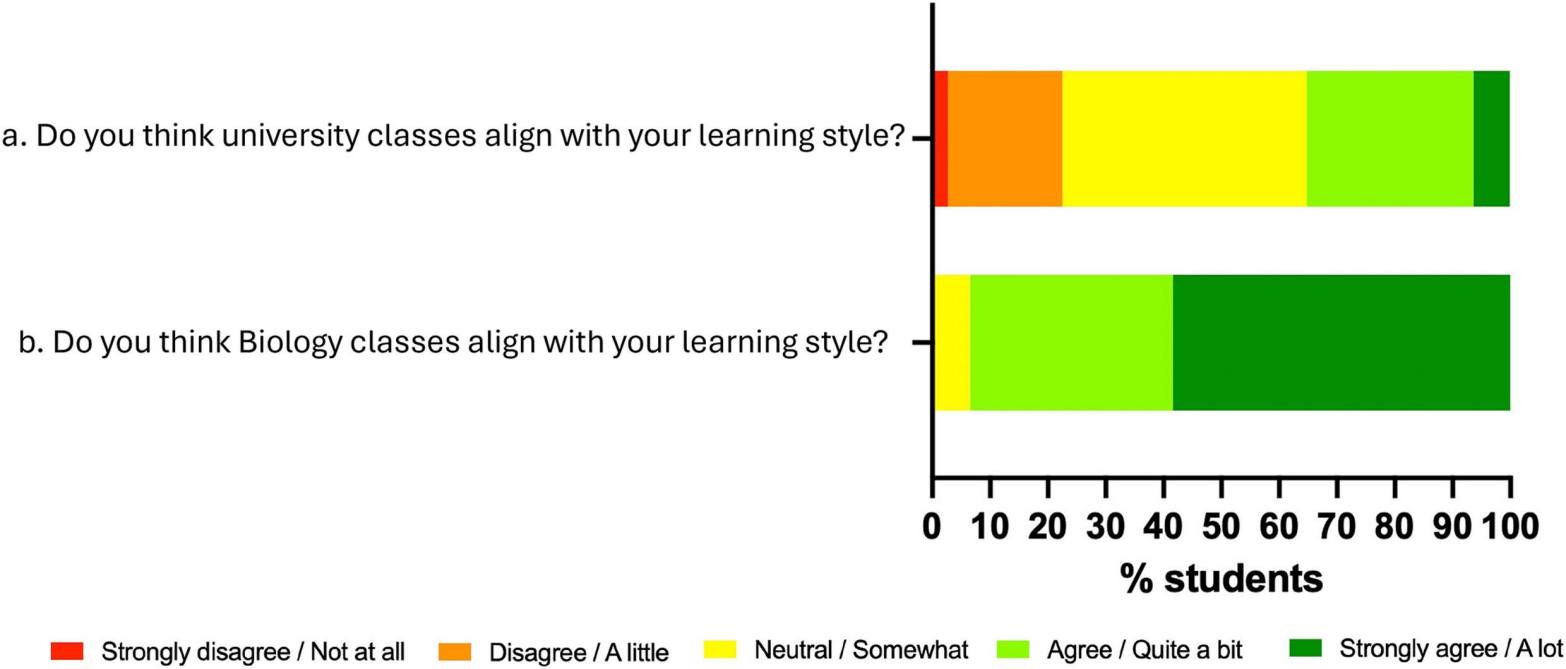
Results of the students’ perception of the biology signature (*n* = 77).

The results indicate a clear increase in positive responses. When asked about all university courses, 34% of students responded favorably (Figure 6a), whereas this percentage rose to 94% when referring specifically to the biology subject (Figure 6b).

## 4 Discussion

The results of this study highlight the effectiveness of implementing diverse gamification strategies to enhance student motivation, engagement, and learning outcomes in the first-year Biology course of the Veterinary degree. Each activity uniquely contributed to reinforcing knowledge, fostering interaction, and promoting a more dynamic learning environment. However, while the findings are promising, several factors must be considered when interpreting the results.

One of the most successful strategies was the integration of Kahoot! as a gamified tool for reviewing key concepts in practical sessions. This approach significantly improved students’ performance in practical exams, demonstrating its effectiveness as a reinforcement tool. Its ease of implementation, minimal time investment (approximately 10 min per quiz), and low cost make Kahoot! a highly accessible strategy for reinforcing key concepts in an interactive manner. However, one limitation of this study is the potential influence of instructor changes during the first year of implementation. Although the structure of practical sessions, presentations, and exams remained unchanged, newly appointed instructors may have also contributed to the observed improvement in student performance. Future studies should control this variable by assessing the long-term impact of Kahoot! across multiple academic years and different instructors.

Another innovative approach involved the adaptation of the classic Who’s Who? game for the Zoology module. This tool proved highly effective in reinforcing complex taxonomic and evolutionary concepts through a strategic and engaging format. Notably, the integration of generative artificial intelligence (GAI) in the development of visual materials provided valuable insights into its applicability in education. While GAI was particularly useful for enhancing low-quality illustrations, its application to high-quality images sometimes resulted in a decline in visual clarity and esthetic appeal. Moreover, survey responses indicated that students highly valued the fact that the game’s illustrations were created by their instructors, underscoring the importance of maintaining a human touch in educational resources.

Beyond gamification, the implementation of dynamic class sessions further contributed to fostering student interaction and engagement. The randomized group formation system encouraged students to collaborate with peers outside their usual social circles, which was particularly beneficial for those who struggle with socialization. This approach not only facilitated teamwork and problem-solving but also contributed to the development of essential soft skills, such as public speaking and critical thinking. These findings align with existing literature suggesting that interactive, student-centered learning environments enhance engagement and improve knowledge retention.

The combination of gamification activities and dynamic class sessions led to a highly positive student perception of the Biology subject. While these findings reinforce the effectiveness of innovative teaching methodologies, it is important to acknowledge other contributing factors, such as the course content, instructional materials, and lecture quality. The Biology subject in this study covers inherently engaging topics, and instructors invest effort into designing visually appealing and scientifically rigorous presentations. Nevertheless, these results support the broader argument for transforming university education to align with contemporary students’ needs. Modernizing teaching strategies by making courses more interactive, engaging, and motivating does not undermine the scientific rigor of higher education; in fact, it enhances knowledge acquisition and fosters a more meaningful learning experience.

## Data Availability

The original contributions presented in the study are included in the article/supplementary material, further inquiries can be directed to the corresponding author.

## References

[1] Putz LM Hofbauer F Treiblmaier H. Can gamification help to improve education? Findings from a longitudinal study. Comput Hum Behav. (2020) 110:106392. doi: 10.1016/j.chb.2020.106392

[2] Santos-Villalba MJ Olivencia JJL Navas-Parejo MR Benítez-Márquez MD. Higher education students’ assessments towards gamification and sustainability: a case study. Sustainability (Switzerland). (2020) 12:1–20. doi: 10.3390/su12208513

[3] Manzano-León A Camacho-Lazarraga P Guerrero MA Guerrero-Puerta L Aguilar-Parra JM Trigueros R. Between level up and game over: a systematic literature review of gamification in education. Sustainability (Switzerland). (2021) 13:1–14. doi: 10.3390/su13042247, PMID: 40225413

[4] Guadaña R. R. H. Bermudez J. R. D. Ramirez E. Q. Tong A. E. U.. Drafting a comprehensive schema for the development of gamified learning application for National University. In ICETC '20: proceedings of the 12th international conference on education technology and computers (2021) (pp. 69–75).

[5] Fernandez-Antolin MM del Río JM Gonzalez-Lezcano RA. The use of gamification in higher technical education: perception of university students on innovative teaching materials. Int J Technol Des Educ. (2021) 31:1019–38. doi: 10.1007/s10798-020-09583-0

[6] Buckley P Doyle E Doyle S. Game on! Students’ perceptions of gamified learning. Educ Technol Soc. (2017) 20:1–10.

[7] Prieto Andreu JM. Una revisión sistemática sobre gamificación, motivación y aprendizaje en universitarios. Teoría De La Educación Revista Interuniversitaria. (2020) 32:73–99. doi: 10.14201/teri.20625

[8] Magnago W Nunes PDC. Gamification and inclusive education: promoting the engagement of all students (2024) 6. doi: 10.56238/arev6n2-147

[9] Dichev C Dicheva D. Gamifying education: what is known, what is believed and what remains uncertain: a critical review. Int J Educ Technol High Educ. (2017) 14:1–36. doi: 10.1186/s41239-017-0042-5, PMID: 40256639

[10] Husnawati Z Carina A. Gamification (Kahoot) and its usage in teaching and learning process for primary education of SD/MI (2023). doi: 10.20961/shes.v6i3.82331

[11] Araújo FJ Gonçalves CC Santos CHAD Da Silva CE. Gamification in education: an analysis of the kahoot! Platform! Revista Ibero-Americana de Humanidades, Ciências e Educação. (2024) 10:246–58. doi: 10.51891/rease.v10i7.14744

[12] Ananda W Halim A. Kahoot! In a flipped classroom: a case study at junior high school in Samarinda. EnJourMe (English Journal of Merdeka): Culture, Language, and Teaching of English. (2024) 9:70–88. doi: 10.26905/enjourme.v9i1.13151

[13] Glenn J. Weinland K.. Cross-disciplinary usage of Kahoot to enhance classroom teaching. In 2024 ASEE midwest section conference (2024).

[14] Kakhkharova M Tuychieva S. AI-enhanced pedagogy in higher education: redefining teaching-learning paradigms (2024) 45:1–6. doi: 10.1109/ickecs61492.2024.10616893

[15] Wang J. Research on the innovative application and effectiveness of AI intelligence in intelligent assisted teaching system for university yoga classroom (2024) 1:64–8. doi: 10.62381/h241312

[16] Hossain R. Prospective artificial intelligence (AI) applications in the university education level: enhancing learning, teaching and administration through a PRISMA base review systematic review. Pak J Life Soc Sci. (2024) 22:9173–91. doi: 10.57239/pjlss-2024-22.2.00694

[17] Donnell F Porter M Rinella Fitzgerald D. The role of artificial intelligence in higher education. Irish J Technol Enhanced Learn. (2024) 8. doi: 10.22554/szwjfy54

[18] Hickman C. P. J. Keen S. L. Eisenhour D. J. Larson A. L’Anson H. (2020). Integrated principles of zoology (18^a^ed.). McGraw-Hill Higher Education.

